# Toxicity and Biodistribution of Fragmented Polypropylene Microplastics in ICR Mice

**DOI:** 10.3390/ijms24108463

**Published:** 2023-05-09

**Authors:** Sijoon Lee, Dongseon Kim, Kyung-Ku Kang, Soo-Eun Sung, Joo-Hee Choi, Minkyoung Sung, Chang-Hoon Shin, Eunyoung Jeon, Dongkyu Kim, Dongmin Kim, Sunjong Lee, Hee-Kyung Kim, Kilsoo Kim

**Affiliations:** 1 Preclinical Research Center, Daegu-Gyeongbuk Medical Innovation Foundation, Daegu 41061, Republic of Korea; sjlee1013@kmedihub.re.kr (S.L.); kds0433@kmedihub.re.kr (D.K.); kangkk@kmedihub.re.kr (K.-K.K.); sesung@kmedihub.re.kr (S.-E.S.); cjh522@kmedihub.re.kr (J.-H.C.); tjdalsrud27@kmedihub.re.kr (M.S.); shinchoon@kmedihub.re.kr (C.-H.S.); duvet111@kmedihub.re.kr (E.J.); dgkim728@kmedihub.re.kr (D.K.); 2 Department of Medical & Biological Engineering, Kyungpook National University, 80 Dahakro, Buk-gu, Daegu 41566, Republic of Korea; 3 Department of Pharmacology, School of Dentistry, Kyungpook National University, 80 Dahakro, Buk-gu, Daegu 41566, Republic of Korea; 4 Korea Institute of Industrial Technology, Chenan 31056, Republic of Koreasunjong1774@kitech.re.kr (S.L.); 5 College of Veterinary Medicine, Kyungpook National University, 80 Dahakro, Buk-gu, Daegu 41566, Republic of Korea

**Keywords:** polypropylene, microplastics, toxicity, biodistribution, Cy5.5-COOH-labeled

## Abstract

Currently, polypropylene (PP) is used in various products, thus leading to high daily exposure in humans. Thus, it is necessary to evaluate the toxicological effects, biodistribution, and accumulation of PP microplastics in the human body. In this study, administration of two particle sizes of PP microplastics (approximately 5 and 10–50 µm) did not lead to any significant changes in several toxicological evaluation parameters, including body weight and pathological examination, compared with the control group in ICR mice. Therefore, the approximate lethal dose and no-observed-adverse-effect level of PP microplastics in ICR mice were established as ≥2000 mg/kg. Furthermore, we manufactured cyanine 5.5 carboxylic acid (Cy5.5-COOH)-labeled fragmented PP microplastics to monitor real-time in vivo biodistribution. After oral administration of the Cy5.5-COOH-labeled microplastics to the mice, most of the PP microplastics were detected in the gastrointestinal tract and observed to be out of the body after 24 h in IVIS Spectrum CT. Therefore, this study provides a new insight into the short-term toxicity, distribution, and accumulation of PP microplastics in mammals.

## 1. Introduction

Globally, plastic usage continues to increase [1]. This excessive use of plastic adds to environmental pollution, thus affecting humankind [2,3,4]. Plastic discarded in the environment is broken into small particles by physical and chemical environmental factors such as weathering, erosion, heat, and ultraviolet rays [5,6,7]. Plastic particles that are smaller than 5 mm are called microplastics [8]. Various microplastics such as polypropylene (PP) and polystyrene (PS) have been detected in the Central Atlantic Ocean in Morocco and the Northwestern Pacific Ocean [9,10]. There are reports of microplastics detected not only in the oceans but also in the soil and atmosphere of Iran [11,12]. Animals living in a microplastic-contaminated environment are exposed to microplastics through inhalation or ingestion [13]. There is much research on the detection of microplastics in living organisms. Microplastics have been detected in mussels, fishes in freshwater, and even in the gastrointestinal tracts of birds [14,15,16,17,18]. Moreover, there have been recent reports of multiple microplastics in human blood [19]. This evidence in the environment, organisms, and humans is directly related to the risk of microplastics. 

Recently, a number of in vitro and in vivo studies have been published to confirm the potential risk of microplastics. Polyethylene (PE) and PS microplastics have been reported to interfere with cell viability, inflammation, and oxidative stress mechanisms. Interestingly, PS caused hepatotoxicity and impaired fat metabolism in liver organoids [20,21]. Additionally, in BEAS-2B, a human bronchial epithelial cell, an increase in cytotoxicity and reactive oxygen species production has been confirmed [22]. These cellular level studies suggest that microplastics primarily affect mechanisms associated with inflammation, oxidative stress, and physiological dysfunction of the system.

Microplastic studies on mammals are mainly conducted using rodents. Microplastics have been detected in the brain of mother mice after oral administration, and behavioral changes and increased autism-related factors in the fetus of mice have been reported [23]. It has been reported that microplastic administration affected flora–metabolite–cytokine axes regulation through hematopoietic system damage [24]. Recently, granulomatous inflammation has been observed in the lungs of mice during repeated oral administration of PE microplastics [25]. It has been reported that microplastic administration affects the expression of microglial differentiation markers along with the activation of NF-κB, thus regulating microglial immune activation in the mouse brain [26]. To assess the health risk of microplastics to the human body, research on microplastics using animals, especially mammals, is increasing. However, most studies are being conducted on specific systems. To overcome this limitation, it is necessary to evaluate the health risks of microplastics through toxicity and pathological analyses of various systems in vivo. In particular, further research on the biodistribution and accumulation of microplastics in vivo is needed. Substances that enter the body from the outside undergo the processes of absorption, distribution, metabolism, and excretion (ADME), and studies on ADME of microplastics are extremely rare [27]. The microplastics that are used to evaluate in vivo biodistribution are mainly spherical, which are labeled with fluorescent materials or tagged with radioactive isotopes [28,29,30]. Some studies have confirmed that spherical labeled or tagged microplastics move toward the liver, kidneys, and genital organs of mice [31,32]. However, microplastics that living organisms are exposed to from the environment have many more fragments or fibers than spherical ones. Therefore, it is necessary to check the biodistribution and accumulation levels by labeling fluorescent materials on microplastics in the form of fragments or fibers. Fluorescent dye labeling is a general strategy for studying the biodistribution of microplastics [33]. 

The combined swelling–diffusion (CSD) synthesis is a common method to prepare fluorescent nanoplastics and microplastics that entails the entrapment of fluorescent materials inside a microplastic by controlling the temperature or solvent combination [34]. The fluorescent dye used in this study, cyanine 5.5 carboxylic acid (Cy5.5-COOH), is commonly used in bioimaging and disease diagnosis due to its excellent spectral properties, including narrow absorption spectrum, and high sensitivity and stability [35]. PP plastics were chosen for this study because of their cost-effectiveness and common and wide usage in daily life [36,37]. They are used for various purposes, including microwave containers, label films for plastic bottles, and paper money [38,39,40]. There are higher chances of PP being exposed to the human body through the oral cavity as it is widely used in food containers and in various objects in everyday life including ropes, twine, tape, upholstery, clothing, camping equipment, etc. In this study, we evaluated the toxicity and biodistribution of two different particle sizes of fragmented PP microplastics in ICR mice. First, two suitable sizes of fragmented PP microplastics were prepared. The approximate lethal dose (ALD) and no-observed-adverse-effect level (NOAEL) were derived by performing a single and 4-week repeated toxicity test recommended by OECD guidelines using the prepared microplastics. Additionally, PP microplastics labeled with Cy5.5-COOH were used to study their biodistribution and accumulation in mice. From these results, we evaluated the toxicity and biodistribution of the PP microplastics in mice.

## 2. Results

### 2.1. Characterization of PP Microplastics

**Figure 1:**
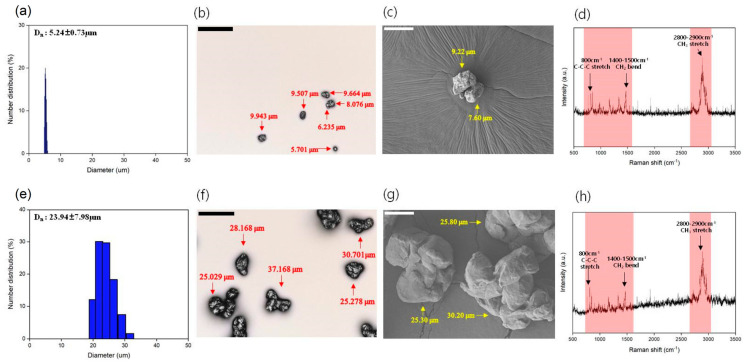
Characterization of prepared polypropylene microplastics with two particle sizes, approximately 5 and 10–50 µm. (**a**,**e**) Average size and distribution of the particle size using a particle size analyzer. (**b**,**c**,**f**,**g**) The actual size of the particles was measured using a 3D profile and SEM. (**d**,**h**) The chemical characters of the particles were verified using Raman spectroscopy. Data are presented as mean ± standard deviation. Scale bars: 50 µm for black and 10 μm for white.

The sizes of particles measured using a particle size analyzer (PSA), confocal, and scanning electron microscopy (SEM) were approximately 5 and 10–50 µm, and the average particle sizes were 5.24 ± 0.73µm and 23.94 ± 7.98µm, respectively, which were fragmented (Figure 1a–c,e–g). Through Raman spectroscopy, the same peak as that of PP plastic raw materials was confirmed for both sizes of the particles (Figure 1d,h).

### 2.2. Single and 4-Week Repeat Toxicity

**Figure 2:**
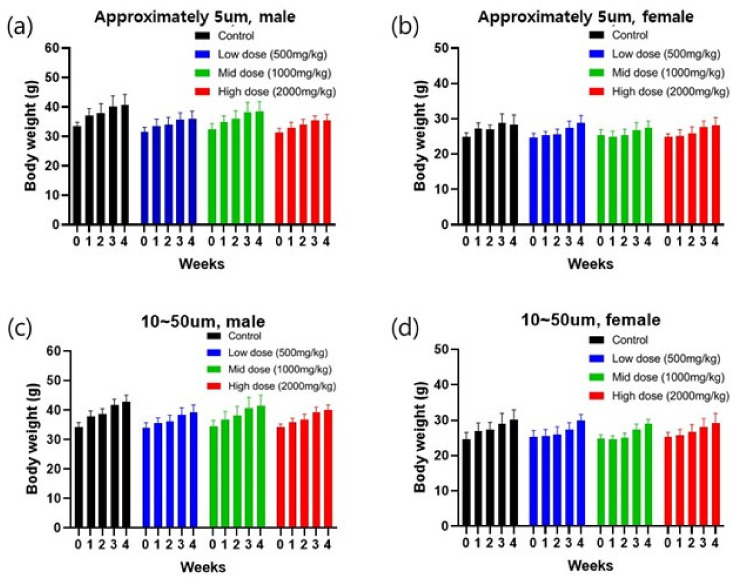
Body weight changes with repeated oral administration of two particle sizes of polypropylene (PP) microplastics for 4 weeks. Body weight changes were measured once a week. (**a**,**b**) Body weight changes of males and females in approximately 5 µm particle size of PP microplastics. (**c**,**d**) Body weight changes of males and females in 10–50 µm size of PP microplastics. Data are presented as mean ± standard deviation.

**Figure 3:**
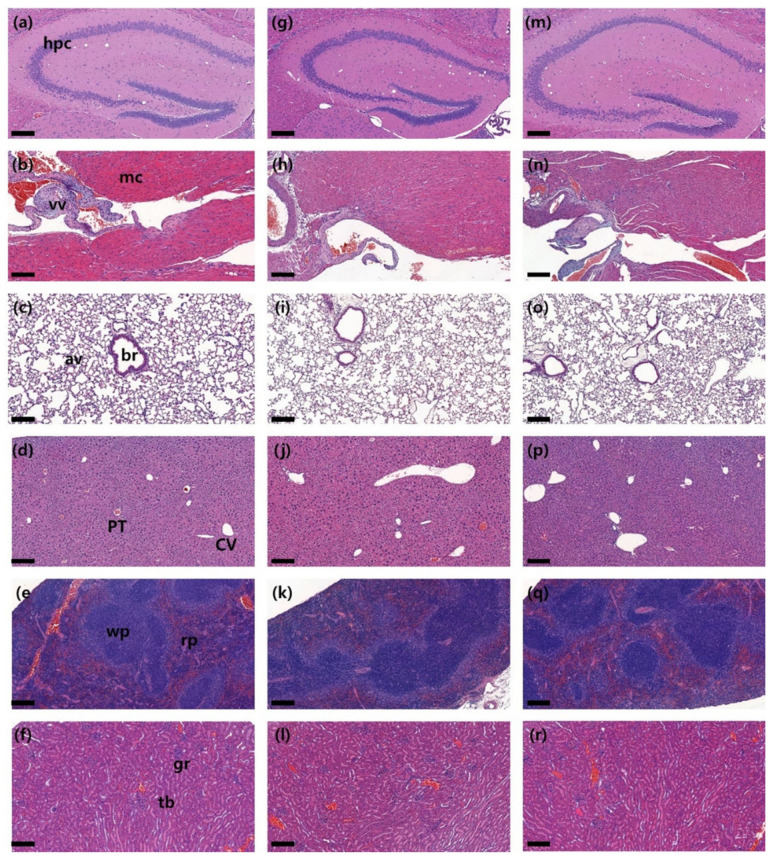
Histopathological evaluation with repeated oral administration of two sizes of polypropylene microplastics for four weeks. Representative picture of histopathological evaluation. Compared to organs of the control group (first line), there were no specific changes in organs of the approximately 5 μm sized PP microplastic–treated group (second line) and the 10~50 μm treated group (third line). (**a**,**g**,**m**) The hippocampus (hpc) of the brain, (**b**,**h**,**n**) myocardium (mc) and valve (vv) of the heart, (**c**,**i**,**o**) bronchiole (br) and alveolar (av) of the lung, (**d**,**j**,**p**) central vein (CV) and portal triad (PT) of the liver, (**e**,**k**,**q**) white (wp) and red (rp) pulp of the spleen, and (**f**,**l**,**r**) glomerulus (gr) and tubules (tb) of the kidney and other organs were evaluated. Scale bar: 200 μm.

During each observation period, no significant differences in clinical signs, food and water consumption, necropsy, postmortem examination, organ weight, hematology and serum biochemistry (Tables S1–S4), body weight changes and histopathological evaluation were observed between the control and PP microplastic-treated groups (Figure 2 and Figure 3). Therefore, the ALD and NOAEL for the two particle sizes of PP microplastics were established to be >2000 mg/kg.

### 2.3. Cy5.5-COOH Labeling of PP Microplastics

**Figure 4:**
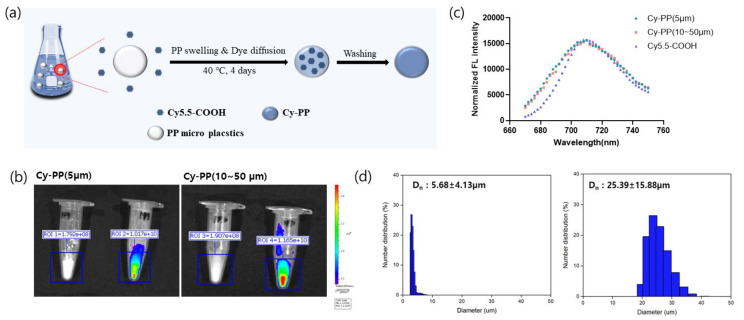
Characterization of Cy5.5-COOH-labeled polypropylene (Cy-PP) microplastics with two particle sizes, approximately 5 and 10–50 µm. (**a**) Synthetic scheme of Cy-PP microplastics. (**b**) In vitro IVIS fluorescence images of both approximately 5 and 10–50 µm particle sizes of Cy-PPs after dye labeling. (**c**) Fluorescence spectra of Cy5.5-COOH and Cy-PP microplastics. (**d**) Average and distribution sizes of the particles using PAS. Data are presented as mean ± standard deviation.

Tetrahydrofuran (THF) was chosen as a good solvent for diffusion of Cy5.5-COOH into PP microplastics by swelling. Distilled water maintains the shape of microplastics and provides a condition of dye diffusion from THF into PP microplastics (Figure 4a). After filtration and washing, Cy5.5-COOH-labeled PP (Cy-PP) microplastics were obtained. In vitro IVIS fluorescence images clearly showed the strong signal from the Cy-PP microplastics of particle size approximately 5 and 10–50 µm (Figure 4b). This strong signal intensity in IVIS images (almost 1 × 10^10^ radiant efficiency/50 mg Cy-PP) enabled Cy-PPs to be applied for in vivo biodistribution study. Moreover, the fluorescence of the Cy-PP microplastics was similar to that of Cy5.5-COOH, exhibiting emission at 710 nm upon excitation at 650 nm (Figure 4c). Because morphology and chemical structure are related to biodistribution and clearance in the body, Cy-PP microplastics were verified using SEM, Fourier transform infrared (FT-IR), and PSA after labeling. SEM images of Cy-PPs showed similar particle size range of unlabeled PP microplastics (Figure S1). The average sizes of Cy-PP microplastics sizes with approximately 5 and 10–50 µm were 5.68 ± 4.13 µm and 25.39 ± 15.88 µm, respectively, with a similar particle sizes to those of unlabeled PP microplastics (Figure 4d). The FT-IR spectrum of the Cy-PP microplastics was also similar to that of the unlabeled PP microplastics (Figure S2). This result indicates that fluorescence labeling did not affect the chemical structures and Cy5.5-COOH was successfully entrapped in PP microplastics. Moreover, the amount of Cy5.5-COOH entering the PP microplastics was measured using a standard curve (Figure S3). Cy5.5-COOH content of 6.8 and 18.7 µg was trapped in 50 mg of Cy-PP microplastics of particle sizes approximately 5 and 10–50 µm, respectively.

### 2.4. In Vitro Stability Study

**Figure 5:**
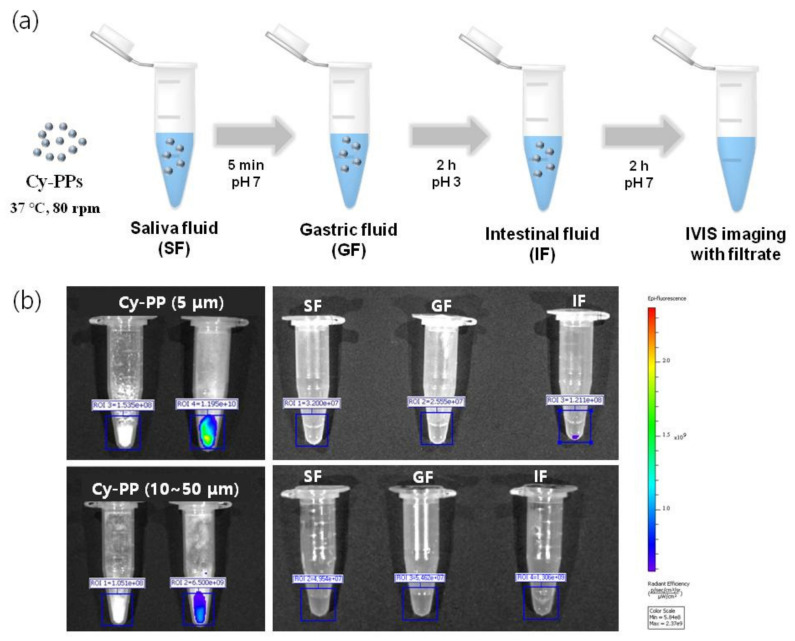
Stability test with Cy5.5-COOH-labeled polypropylene (PP) microplastics in simulated gastrointestinal digestion. (**a**) Procedure of simulated gastrointestinal digestive system. (**b**) In vitro fluorescence images of the filtrate from gastrointestinal digestion simulation.

The fluorescence stability of the Cy-PP microplastics dispersed in corn oil was evaluated for 10 min by measuring the change in signal intensity to confirm the entrapment of Cy-PP microplastics (Figure S4). The fluorescence intensities of all three samples (Cy5.5-COOH, Cy-PP (approximately 5 µm), and Cy-PP (10–50 µm)) were maintained over 95% for 10 min of exposure. Furthermore, in vitro digestion process simulation was performed before in vivo biodistribution study to prove the stability of Cy-PP microplastics in the body [41]. The simulated digestion consisted of three solutions: salivary, gastric, and intestinal fluids (SF, GF, and IF, respectively). Cy-PP microplastics underwent the digestive processes with three phases of simulated digestive system by following a known method (Figure 5a) [42]. No significant release of Cy5.5-COOH was observed with little fluorescence signal from each digestive fluid according to serial digestive steps (Figure 5b).

### 2.5. Biodistribution Study of Cy-PP Microplastics

**Figure 6:**
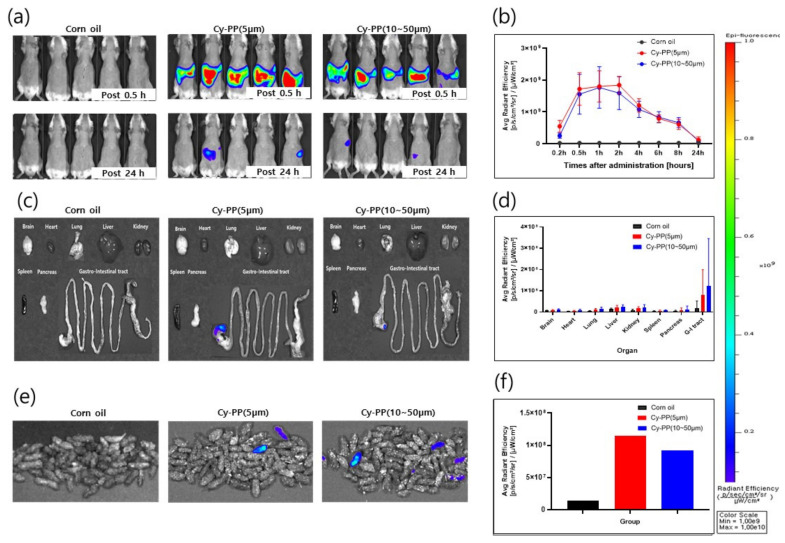
Evaluation of in vivo and ex vivo biodistribution using Cy5.5-COOH-labeled PP (Cy-PP) microplastics. (**a**) Fluorescence images of ICR mice with oral administration of corn oil, Cy-PP (approximately 5 µm), and Cy-PP (10–50 µm) at 0.5 and 24 h. (**b**) Fluorescence signals intensity of the entire body of ICR dependent on time. (**c**,**d**) Ex vivo images and fluorescence intensities for each organ of Cy-PP microplastics-administered ICR mice at 24 h. (**e**,**f**) Fluorescence images and intensity graphs of feces collected 24 h after Cy-PP administration.

Figure 6 and Figure S5 show the representative IVIS Spectrum data at 0.2, 0.5, 1, 2, 4, 6, 8, and 24 h after oral administration of PP or Cy-PP microplastics. Both Cy-PP microplastic sizes of approximately 5 and 10–50 µm were mostly observed from 30 min to 2 h after oral administration (Figure 6b). However, the fluorescence signal intensity was decreased after 4 h of administration for both particle sizes of Cy-PP microplastics, and the corn oil treatment group presented a similar fluorescence signal intensity at 24 h after administration (Figure 6a,b). In the ex vivo distribution evaluation, each organ was harvested by animal sacrifice at 24 h after administration. Higher fluorescence signals were detected in the gastrointestinal tract of mice in the Cy-PP microplastic-administered groups compared with that in the corn oil-administered group as well as other organs (Figure 6c,d). It was confirmed that Cy-PP was excreted by the gastrointestinal tract through feces by measuring the fluorescence signal of the feces collected at 24 h after administration in both size of Cy-PP-administered groups (Figure 6e,f). There were no significant differences in other organ distributions and excretion pathways according to particle size and gender differences.

## 3. Discussion

As the amount of used plastic increases, the amount of discarded plastic is also increasing. Some of the discarded plastics are recycled, but nonrecycled plastics turn into microplastics, plastic particles of <5 mm, due to various environmental factors [5,6,7,8]. The generated microplastics primarily pollute the environment, such as the soil, ocean, and atmosphere, and cause ecological pollution due to direct exposure to the organisms living in that environment or ingestion [3,13,43,44,45,46,47,48]. It can then be exposed to the human body by the food chain [49]. Exposure of microplastics through food is also expected to affect humans. Previously, it was reported that 20 microplastics have been detected per 10 g in the feces of eight people, and a total of nine types of microplastics, including the most-detected PP and PE terephthalate [50]. In particular, 184 microplastics have been detected per liter in wine, and there are reports that people ingest a single plastic credit card amount of microplastics a week [51,52]. Many studies have recently been reported to identify the potential risks of microplastic exposure to living organisms and humankind. At the cellular level, the treatment of PS in the Caco-2 cell resulted in the increased cell uptake and inhibited the activity of mitochondrial depolarization and adenosine triphosphate-binding cassette transport, thus causing disruption to the cellular efflux and defense system [53]. The growth and reproduction of *Hyalella azteca* decreased after exposure to microplastics, and in zebrafish, dysfunction of the oogenesis process and neurotoxicity were induced by microplastics [54,55]. Previous microplastic studies reported lower sperm count, decrease in weight of testicles, increased deformity, abnormal sperm ratio, and collapse of testicular normal structure in male mice [29,56]. In female mice, presence of microplastics in the ovary, decrease in the number and size of the growing follicle, and increase incidence of ovarian fibrosis have been observed [57]. Additionally, changes in the immune system have been reported due to a decrease including Treg cell., by inducing changes in intestinal microbiome [58]. 

Recent reports suggest that PP microplastics are one of the most used and abundant in human daily life [37]. Because there is a high possibility that they can affect the human body, it is necessary to evaluate the toxicity and accumulation of microplastics in various organs. The two particle sizes of PP microplastics that were manufactured (Figure 1) in this study did not cause significant toxicological changes compared with the control group in several parameters, including body weight and pathological examination (Figure 2 and Figure 3). Therefore, the two particle sizes of PP microplastics were determined to be nontoxic in the single and 4-week repeated toxicity tests. Thus, the ALD and NOAEL of PP microplastics were indicated to be >2000 mg/kg. To visualize and prove the potential risks of microplastics, research on the ADME of microplastics is also drawing attention. In a recent study, after administering the labeled microplastics to zebrafish, microplastics have been identified in the form of fragments and were detected in the gut [59]. In mice, 29.4% of administered labeled microplastics were also detected in the brain [23]. We labeled the two particle sizes of PP microplastics with fluorescent dye Cy5.5-COOH. After oral administration of the labeled microplastics to the mice, most of labeled microplastics were observed in the gastrointestinal tract. Moreover, most of labeled microplastics were observed to be out of the body after 24 h in IVIS Spectrum CT. We observed that in the extracted organs, some microplastics partially remained in the gastrointestinal tract and were not observed in other organs. From the aforementioned results, it provides a new insight on the toxicological significance of two particle sizes of PP microplastics, along with the distribution and accumulation levels of PP microplastics in mammals. For future studies, long-term administration of microplastics with higher doses than the doses in this study and analysis of additional organs should be considered.

## 4. Materials and Methods

### 4.1. Materials and Reagent

Tetrahydrofuran (THF) and ethyl alcohol (anhydrous, 99.9%) were purchased from Sigma–Aldrich (St. Louis, MO, USA). Cyanine 5.5 carboxylic acid (Cy5.5-COOH) was purchased from Lumiprobe Ltd. (Wan Chai, Hong Kong). For the stability test with the digestive system, three kinds of simulated solutions, salivary, gastric, and intestinal fluids (SF, GF, and IF) were prepared using a previously described method [41]. 

### 4.2. Animals and Ethics Statement

Five-week-old ICR mice, 119 male and 119 female, specific pathogen-free (KOATECH Inc., Pyeongtaek, Republic of Korea), were purchased. During the experimental period, the animals were housed under standard specific pathogen-free facility and kept in environmental conditions with ventilated IVC (395W × 346D × 213H) at a temperature of 22 °C ± 1 °C, relative humidity of 50% ± 10%, ventilation time of 10–15 h, light for 12 h/day, and an illumination of 150–300 lux. All mice care and experimental procedures were approved by the Institutional Animal Care and Use Committee of the Preclinical Center of the KMEDI hub (IACUC; approval No. KMEDI-22020302-00) and were in accordance with their guidelines.

### 4.3. Manufacture of PP Microplastics

PP beads (PropylTex^®^ 50, Micro Powders Inc., New York, NY, USA), a raw material, were ground to prepare PP microplastics in two different particle sizes, approximately 5 µm and 10–50 µm. Briefly, to manufacture PP microplastics with a particle size of 10–50 µm, PP beads were completely frozen at −78 °C and then ground with a blade-shaped homogenizer for 4 to 5 h to form a powder. Then, the powder was filtered through a 10 and 63 µm mesh filter, followed by washing 4 to 5 times in ethanol. Then, it was subjected to drying at 50 °C for 48 h. To manufacture PP microplastics with particle size approximately 5 µm, 10 to 50 µm PP microplastics were dispersed in ethanol and were ground using a high-pressure homogenizer (four pass with 600 bars). Then, the ground particles were filtered with 5 and 15 µm mesh filters and washed 4 to 5 times with ethanol, followed by drying at 50 °C for 48 h.

### 4.4. Characterization of PP Microplastics

The average particle size of microplastics was measured using PSA (ELS-Z2Plus, Otsuka, Japan), and the actual shape and particle size were confirmed by SEM (JSM-6701F, JEOL Inc., Akishima, Tokyo, Japan) and 3D profile (Confocal Microscopy, Keyence, IL, USA). The chemical properties of the particles were analyzed using Raman spectroscopy (RAMANtouch, Nanophoton, Osaka, Japan). Briefly, after exploring the morphology of particles with a 20× objective lens, Raman spectra were collected in the range of 160–3000 cm^−1^ using 300 lines per millimeter grid with a 50 µm slit width. The spectrum of particles was measured over a 16-bit dynamic range with Peliter-cooled charge-coupled element detectors. The acquisition time and accumulation number were adjusted for each scan to obtain enough signals for conducting a library search. The spectrometer was calibrated with silicon at a line of 520.7 cm prior to obtaining the spectrum. Raw Raman spectra underwent noise reduction by polynomial baseline correction and vector normalization to enhance spectral quality (LabSpec 6 software, Horiba Scientific, Kyoto, Japan). The Raman spectra were compared with those of the SLoPP Library of Microplastics and the spectral library KnowItAll software (Bio-Rad Laboratories, Inc., Hercules, CA, USA). Similarities above the hit quality index of 80 were thought to be satisfactory.

### 4.5. Fluorescence Labeling of PP Microplastics

PP microplastics were fluorescently labeled using the CSD method for biodistribution analysis [33]. First, 15 g of PP microplastics were added to 150 mL of distilled water and 150 mL of THF and stirred for 10 min. A Cy5.5-COOH solution (50 mg mL^−1^) in DMSO of 0.5 mL was added to the PP microplastic suspension followed by stirring for 4 days. After the reaction, the Cy-PP microplastics were separated by vacuum filtration using a qualitative filter paper (2 to 3 µm) to remove unlabeled Cy5.5-COOH in the reaction suspension and then washed with distilled water and ethyl alcohol. The Cy-PP microplastics were dried in a dark place at 40 °C. The morphology and chemical structure of the Cy5.5-labeled PP were analyzed using SEM and FT-IR spectroscopy, respectively.

### 4.6. Single Toxicity Test

A single oral administration toxicity test was performed to observe the toxic reactions of two particle sizes of PP microplastics with a single oral administration and to confirm the ALD. Twelve male and female 6-week-old ICR mice were separated into the control, low-dose (500 mg/kg), medium-dose (1000 mg/kg), and high-dose (2000 mg/kg) groups for two different particle sizes of PP microplastics. Corn oil was administered to the control group, and PP microplastic suspended in corn oil at a dosage of 10 mL/kg liquid amount were administered to the other groups as a single oral administration. During the 2-week period, clinical sign observation (once a day), morbidity or dead animal observation (twice a day), and body weight measurement (once a week) were performed. After the end of the observation period, all animals were euthanized using carbon dioxide anesthesia and exsanguinated via the abdominal aorta, followed by necropsy and gross postmortem examination. All conditions for the experiment were set with reference to the OECD Test Guidelines 423 [60].

### 4.7. Four-Week Repeated Toxicity Test

A 4-week repeated oral administration toxicity test was conducted to evaluate the toxicity response and safety of PP microplastics. Forty male and female 6-week-old ICR mice were separated into the control, low-dose (500 mg/kg), medium-dose (1000 mg/kg), and high-dose (2000 mg/kg) groups. PP microplastic suspension in corn oil at a dosage of 10 mL/kg was orally administered to all the groups except the control group once a day for 4 weeks. During the 4-week observation period, clinical sign and morbidity or dead animal observations were performed once and twice a day, respectively. Body weight and food and water consumption were measured once a week. After the end of the observation period, blood was collected via the abdominal aorta under isoflurane anesthesia. A blood cell analyzer (ADVIA 2120i, SIEMENS, Munich, Germany) and a serum biochemistry analyzer (TBA 120-FR; Toshiba, Japan) were used to perform hematological and hematochemical analyses, respectively. Complete gross postmortem examinations were performed on all animals and tissues. The adrenal gland, brain, cecum, colon, duodenum, epididymis, esophagus, heart, ileum, jejunum, kidney, liver, lungs, ovary, pancreas, parathyroid gland, pituitary gland, rectum, spinal cord, spleen, stomach, testis, thymus, thyroid gland, trachea, and uterus were harvested. Among the extracted organs, the brain, spleen, heart, kidney, liver, testis, epididymis, and ovary were weighed. All extracted organs were fixed to 10% neutral-buffered formalin. For histopathological evaluation, a tissue processor (Thermo Fisher Scientific, Inc., Runcorn, UK) was used for procedure of the tissues from the formalin-fixed samples. The paraffin-embedded tissue blocks were cut to a 4 µm thickness and mounted onto glass slides. Staining was performed with hematoxylin and eosin using an autostainer (Dako Coverstainer; Agilent, Santa Clara, CA, USA). The histopathological evaluation of all the slides was performed in a blind manner.

### 4.8. In Vivo Biodistribution Study of Cy-PP Microplastics in Mice

Fifteen male and female 6-week-old ICR mice were separated into the control, Cy-PP (approximately 5 µm), and Cy-PP (10–50 µm) groups. ICR mice had their hair removed to minimize autofluorescence generated from the hair and were fasted 8 h before fluorescence imaging. Two particle sizes of Cy-PPs were orally administered to mice as a solution dispersed in corn oil at a concentration of 2000 mg/kg. Fluorescent images were acquired by the IVIS Spectrum CT (PerkinElmer, Waltham, MA, USA) at excitation and emission wavelength of 675 and 720 nm, respectively. In vivo images were acquired under 2.5% isoflurane anesthesia, and imaging process was performed at 0.2, 0.5, 1, 2, 4, 6, 8, and 24 h after administration. Twenty-four hours after oral administration of corn oil for control, Cy-PP (approximately 5 µm), and Cy-PP (10–50 µm), feces were collected from mice in group cages, and in vitro evaluation was performed. At 24 h after administration, in vivo imaging was the end point, saline perfusion was performed via the left ventricle of the mouse before sacrifice, and ex vivo imaging was performed.

### 4.9. Statistics

All data were presented as mean ± standard deviation. The statistical significance of the differences between the treated and control groups was evaluated by a Student’s *t*-test using the SAS program (version 9.4 SAS Institute Inc., Cary, NC, USA).

## Data Availability

The data that support the findings of this study are available from the corresponding author upon reasonable request.

## Supplementary Materials

The supporting information can be downloaded at: https://www.mdpi.com/article/10.3390/ijms24108463/s1.
