# Supplementary material for: Toxicity and Biodistribution of Fragmented Polypropylene Microplastics in ICR Mice
Source: Int J Mol Sci. 2023 May 9;24(10):8463. doi: 10.3390/ijms24108463 (PMC10218338; doi:10.3390/ijms24108463)
Supplement: Supplementary file 1 [file ijms-24-08463-s001.zip › ijms-2309475-supplementary.pptx]

## Slide 1
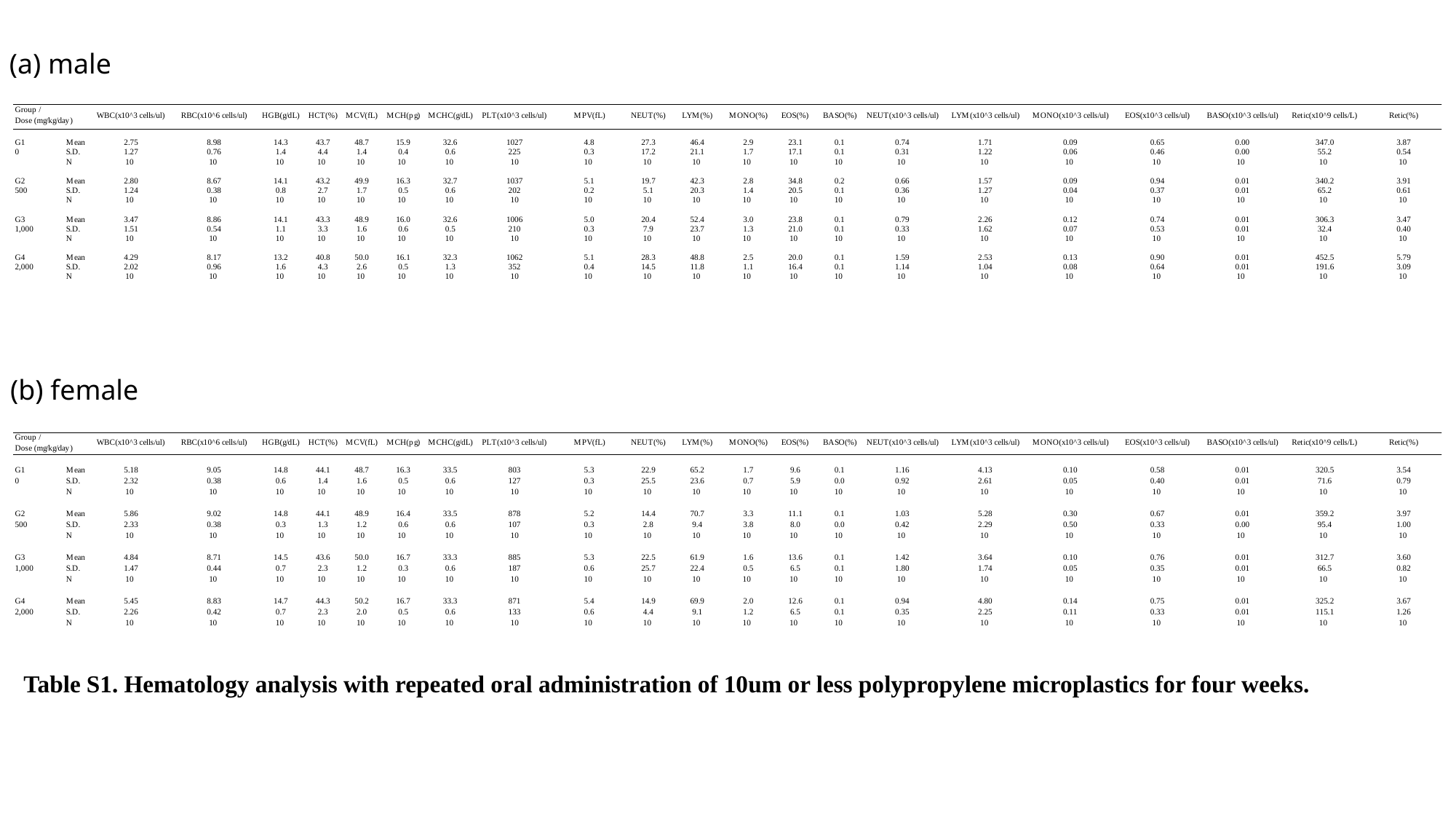

(a) male
(b) female
Table S1. Hematology analysis with repeated oral administration of 10um or less polypropylene microplastics for four weeks.

## Slide 2
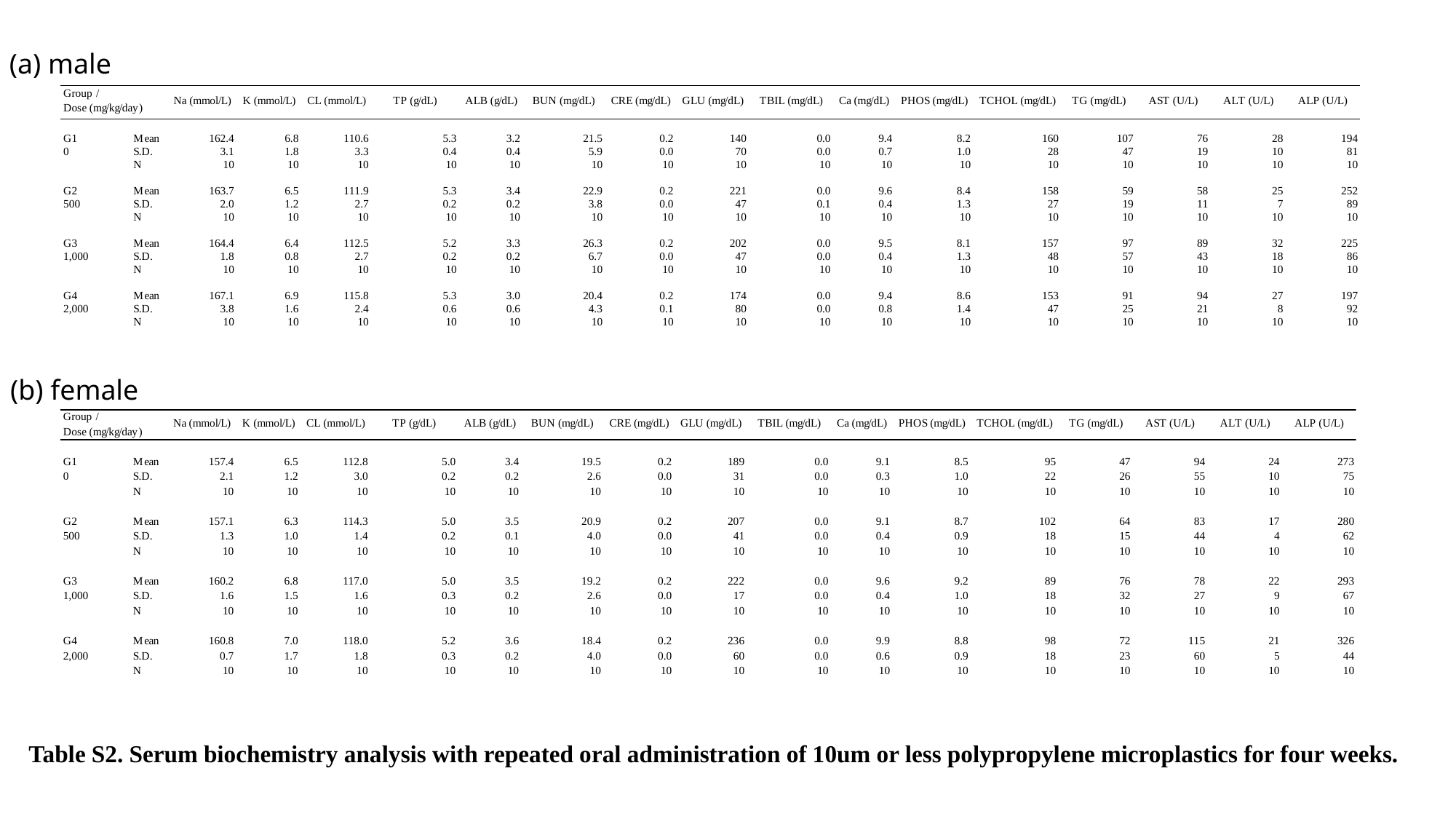

(a) male
(b) female
Table S2. Serum biochemistry analysis with repeated oral administration of 10um or less polypropylene microplastics for four weeks.

## Slide 3
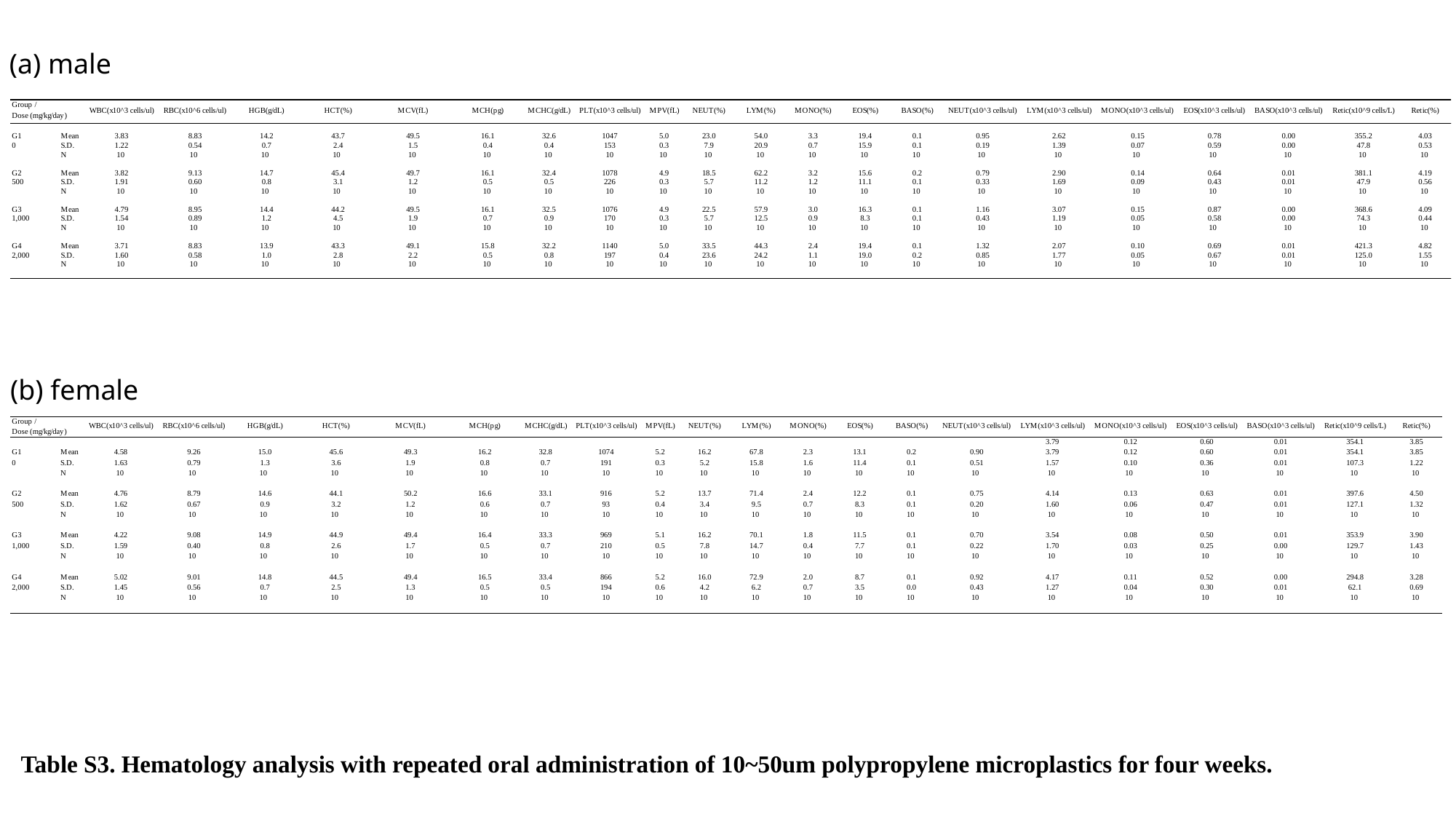

(a) male
(b) female
Table S3. Hematology analysis with repeated oral administration of 10~50um polypropylene microplastics for four weeks.

## Slide 4
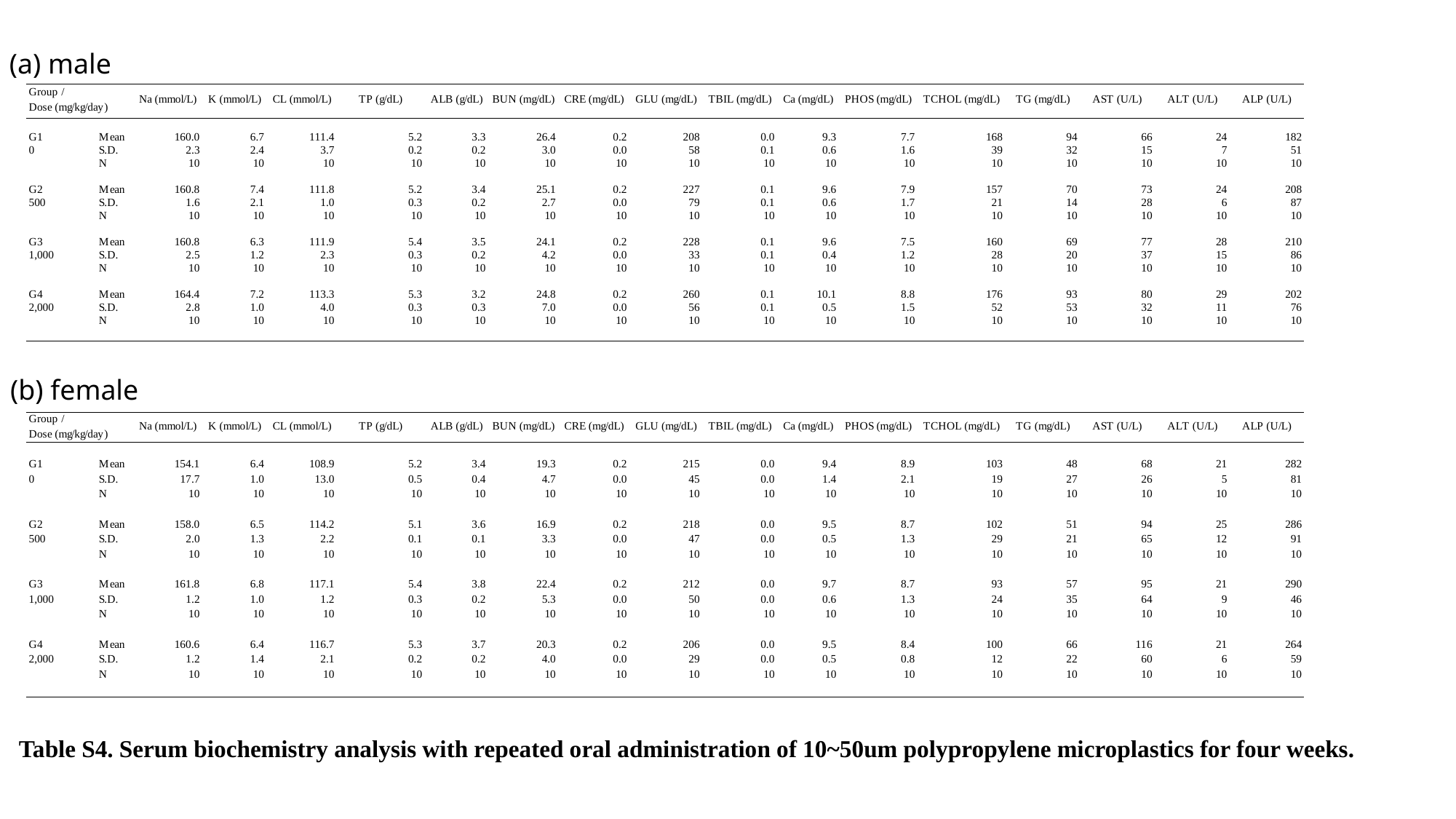

(a) male
(b) female
Table S4. Serum biochemistry analysis with repeated oral administration of 10~50um polypropylene microplastics for four weeks.

## Slide 5
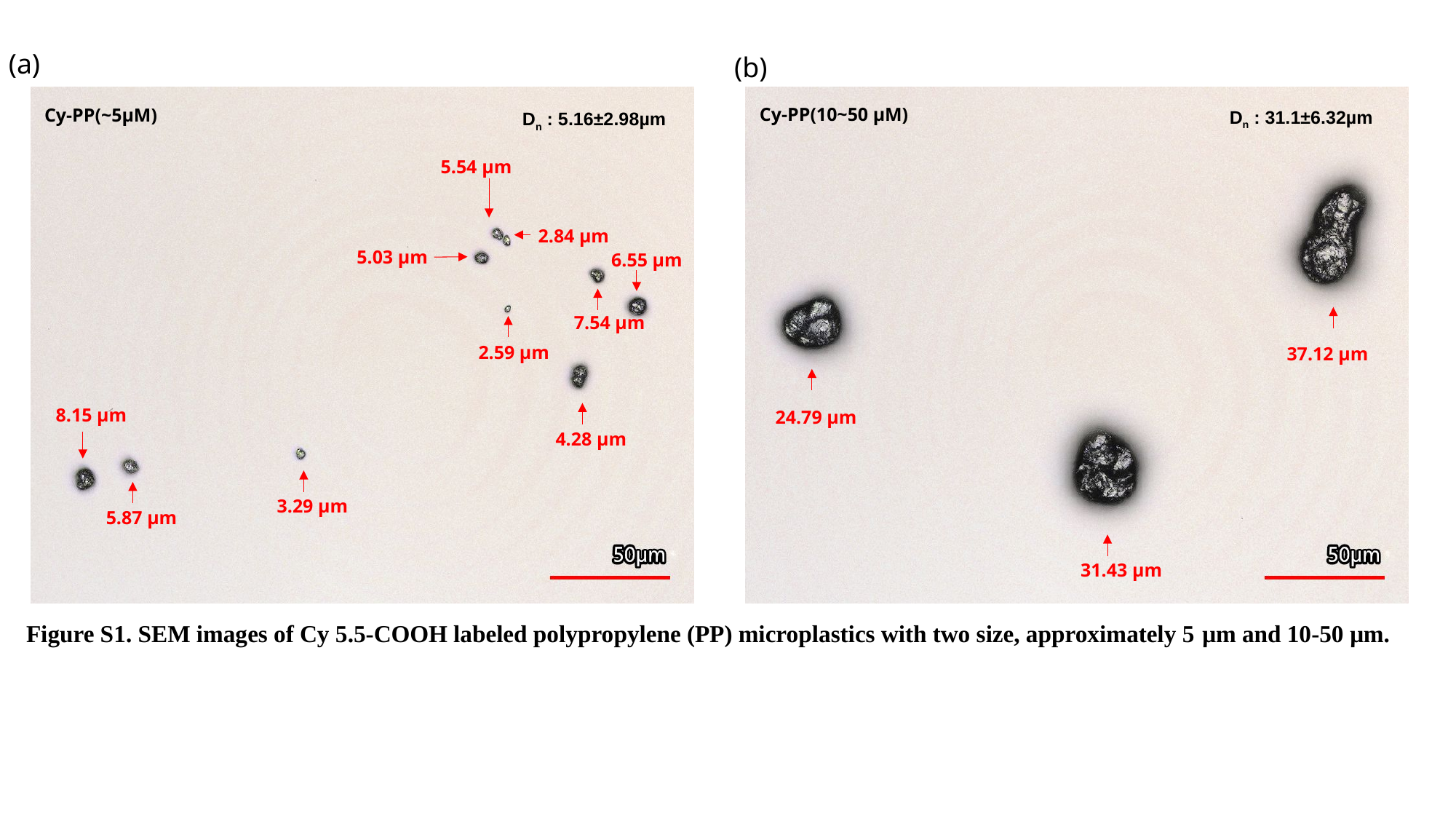

(a)
(b)
Dn : 5.16±2.98µm
5.54 μm
2.84 μm
5.03 μm
6.55 μm
7.54 μm
2.59 μm
8.15 μm
4.28 μm
3.29 μm
5.87 μm
Dn : 31.1±6.32µm
37.12 μm
24.79 μm
31.43 μm
Cy-PP(10~50 µM)
Cy-PP(~5µM)
Figure S1. SEM images of Cy 5.5-COOH labeled polypropylene (PP) microplastics with two size, approximately 5 μm and 10-50 μm.

## Slide 6
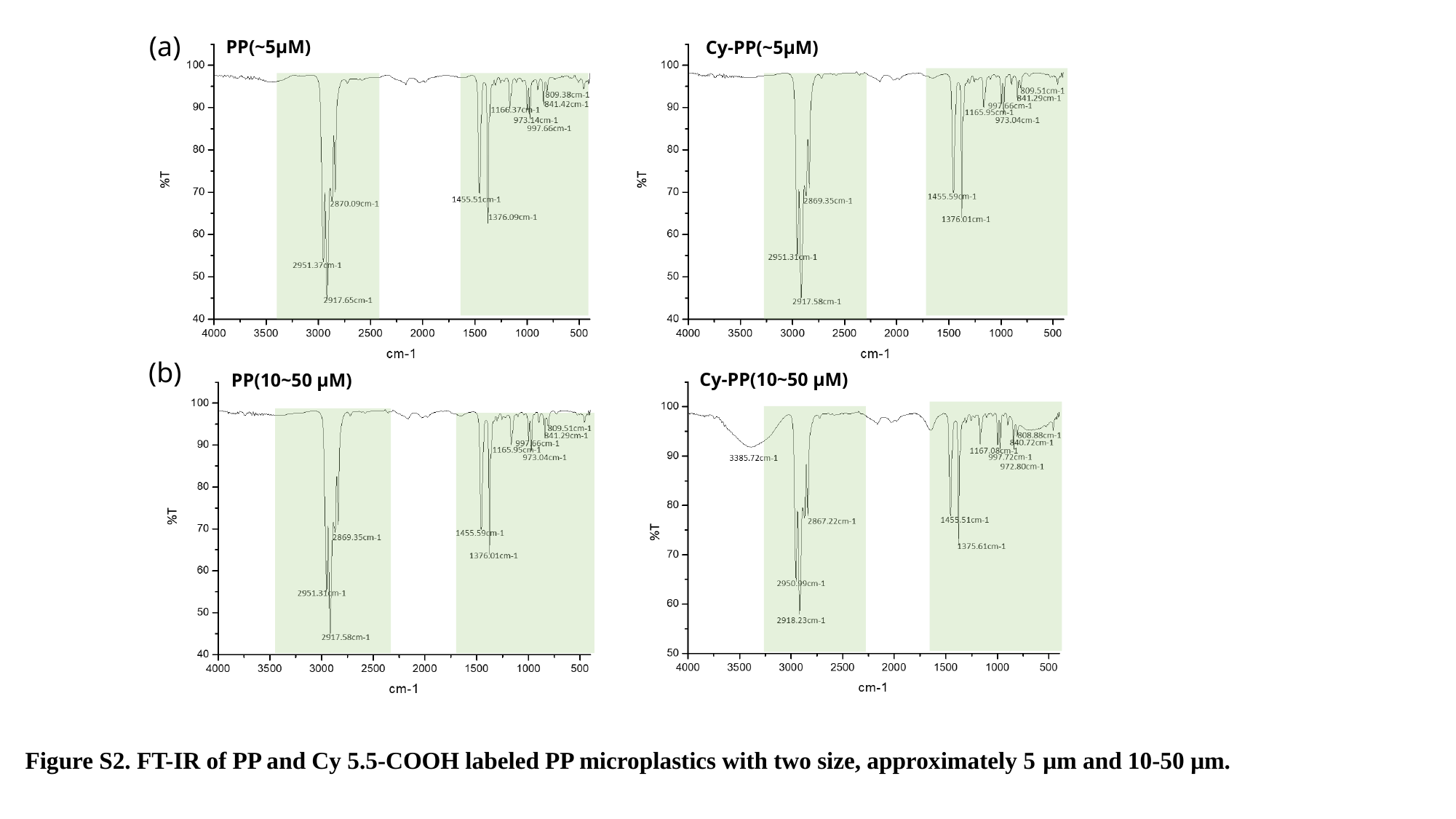

(a)
PP(~5µM)
Cy-PP(~5µM)
(b)
Cy-PP(10~50 µM)
PP(10~50 µM)
Figure S2. FT-IR of PP and Cy 5.5-COOH labeled PP microplastics with two size, approximately 5 μm and 10-50 μm.

## Slide 7
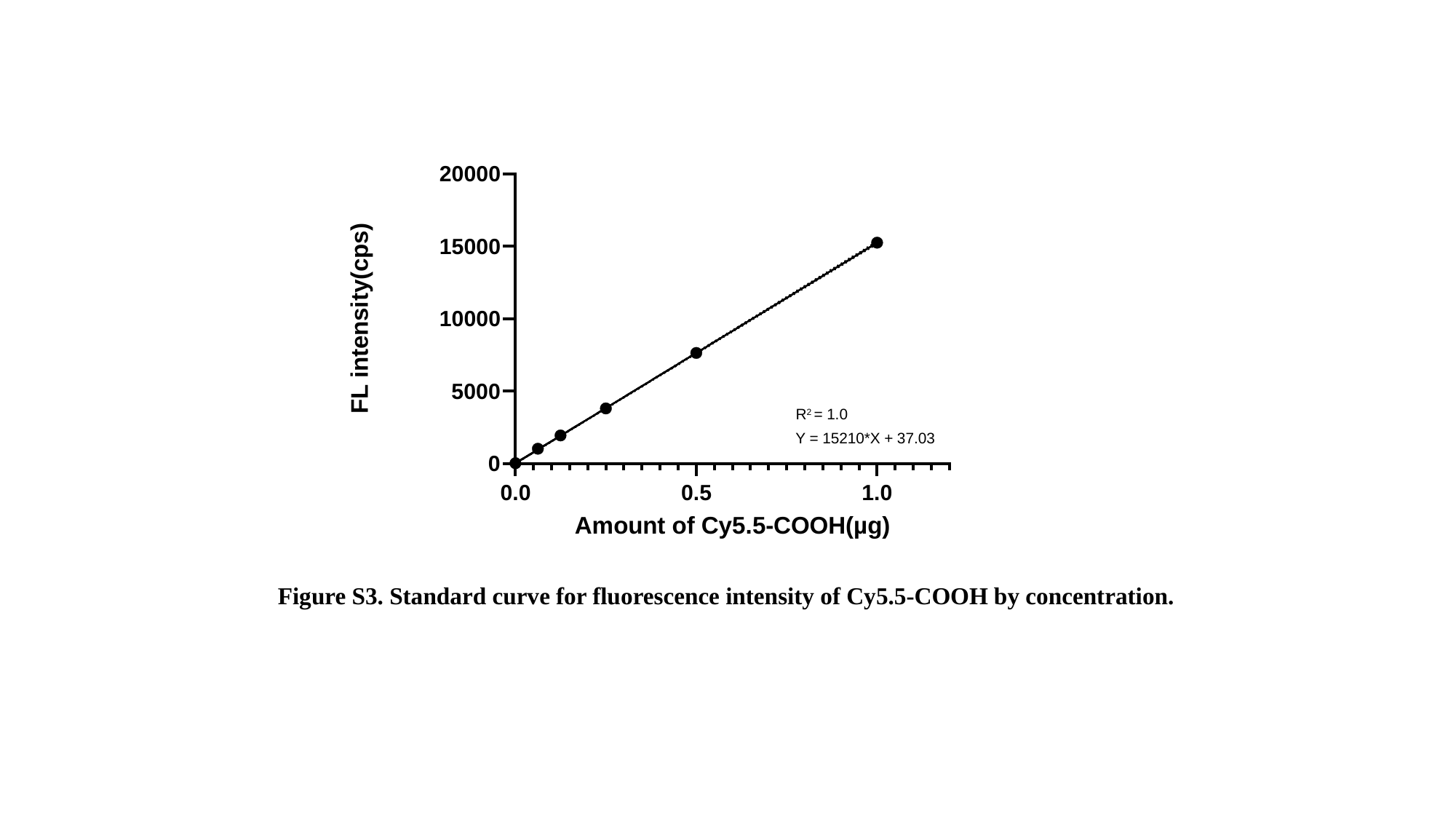

| R2 = 1.0 | |
| --- | --- |
| Y = 15210\*X + 37.03 |
| --- |
Figure S3. Standard curve for fluorescence intensity of Cy5.5-COOH by concentration.

## Slide 8
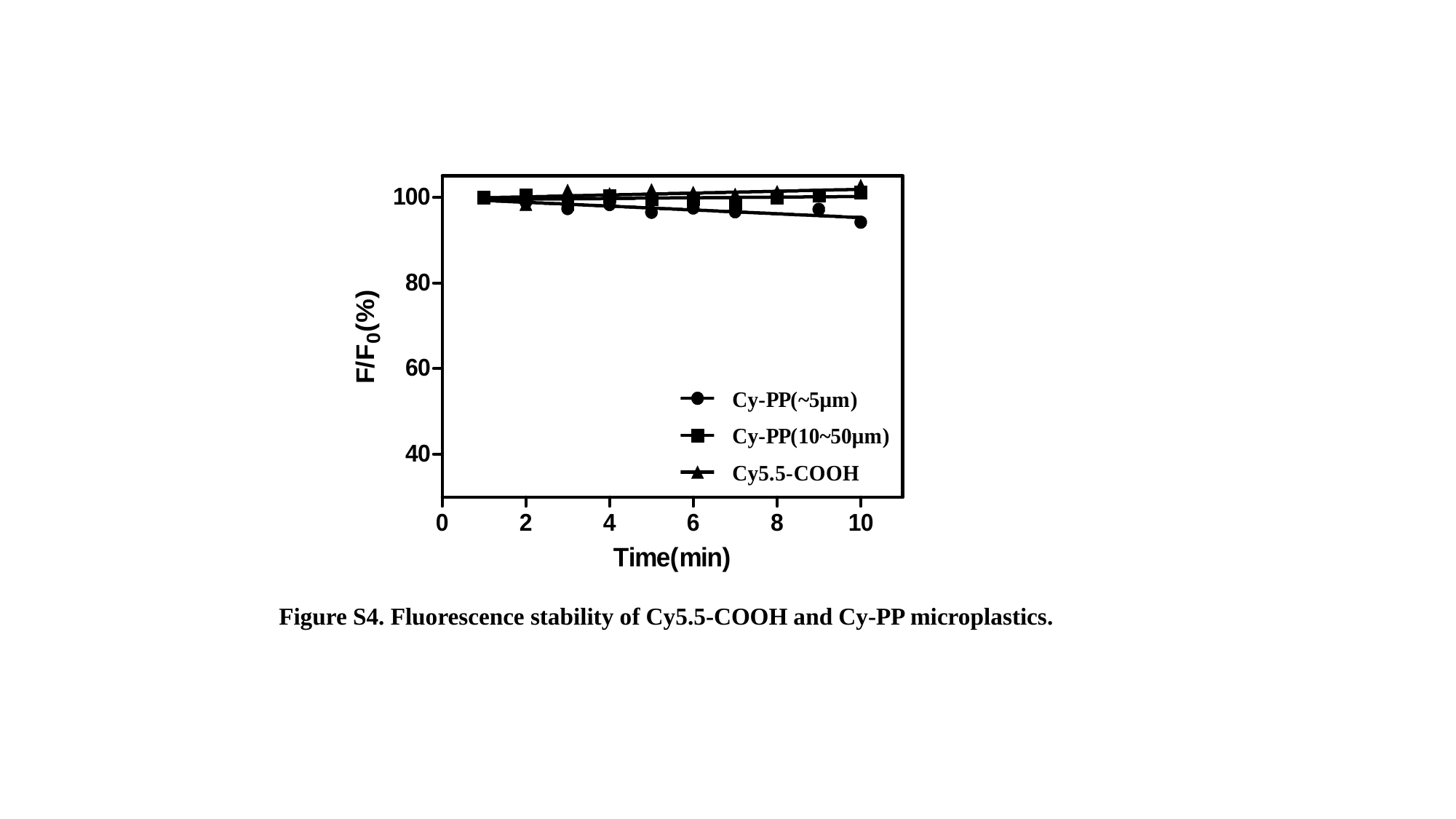

Figure S4. Fluorescence stability of Cy5.5-COOH and Cy-PP microplastics.

## Slide 9
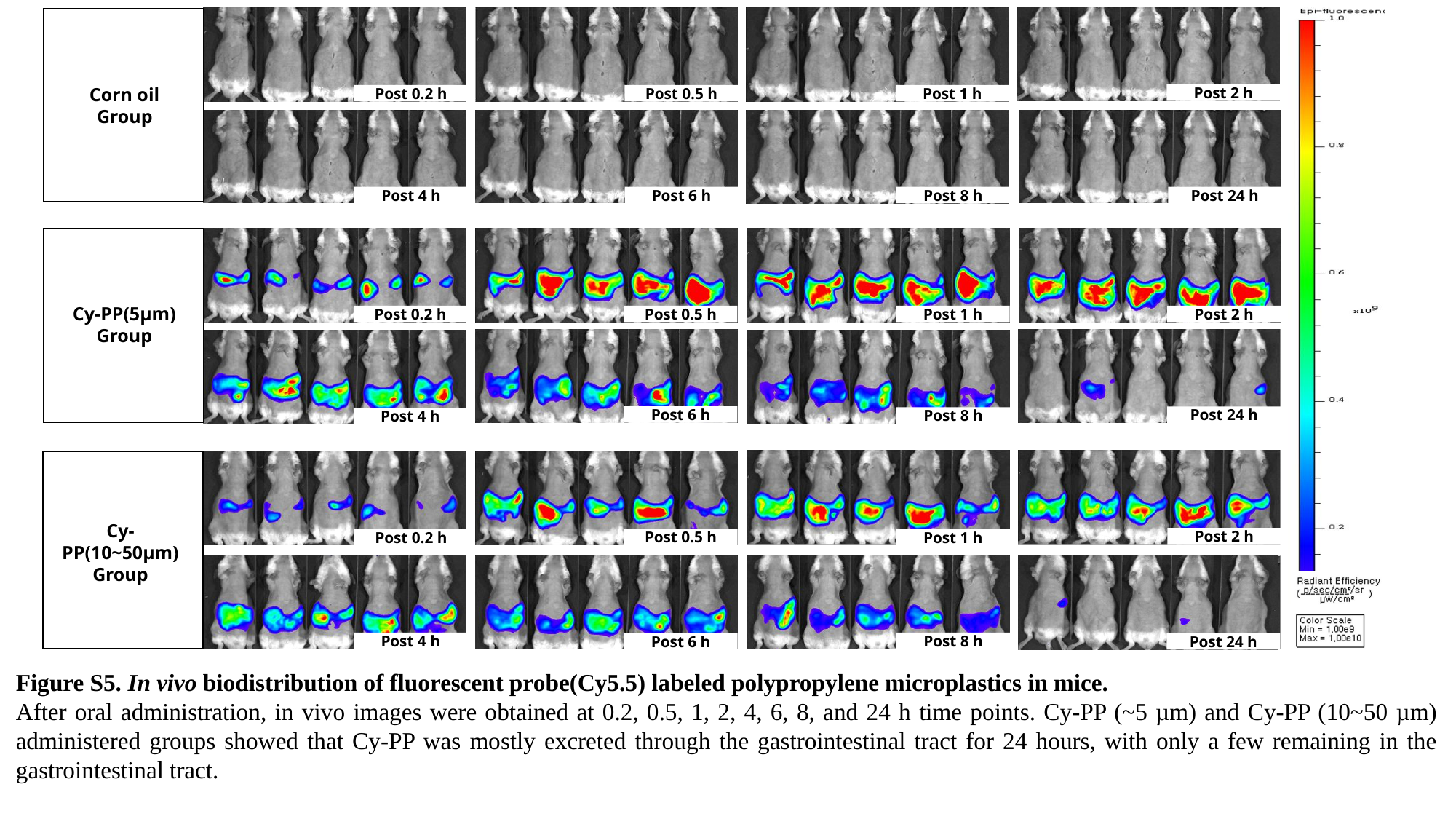

Post 2 h
Post 1 h
Post 0.2 h
Post 0.5 h
Corn oil
Group
Post 8 h
Post 24 h
Post 4 h
Post 6 h
Post 1 h
Post 2 h
Post 0.2 h
Post 0.5 h
Cy-PP(5µm)
Group
Post 6 h
Post 24 h
Post 8 h
Post 4 h
Post 2 h
Post 0.5 h
Post 1 h
Post 0.2 h
Cy-PP(10~50µm)
Group
Post 8 h
Post 4 h
Post 24 h
Post 6 h
Figure S5. In vivo biodistribution of fluorescent probe(Cy5.5) labeled polypropylene microplastics in mice.
After oral administration, in vivo images were obtained at 0.2, 0.5, 1, 2, 4, 6, 8, and 24 h time points. Cy-PP (~5 µm) and Cy-PP (10~50 µm) administered groups showed that Cy-PP was mostly excreted through the gastrointestinal tract for 24 hours, with only a few remaining in the gastrointestinal tract.
